# Improving acetyl-CoA biosynthesis in *Saccharomyces cerevisiae* via the overexpression of pantothenate kinase and PDH bypass

**DOI:** 10.1186/s13068-017-0726-z

**Published:** 2017-02-17

**Authors:** Wenshan Liu, Bo Zhang, Rongrong Jiang

**Affiliations:** School of Chemical & Biomedical Engineering, Nanyang Technological University, 62 Nanyang Drive, Singapore, 637459 Singapore

**Keywords:** Acetyl-CoA, Pantothenate kinase, Pyruvate dehydrogenase bypass, Naringenin production, Pantothenate, *Saccharomyces cerevisiae*

## Abstract

**Background:**

Acetyl-CoA is an important precursor in *Saccharomyces cerevisiae*. Various approaches have been adopted to improve its cytosolic level previously with the emphasis on engineering the “acetyl-” part of acetyl-CoA. To the best of our knowledge, there have been no reports on engineering the “-CoA” part so far.

**Results:**

In this study, we had tried to engineer *S. cerevisiae* from both the “-CoA” part via pantothenate kinase overexpression (PanK from *S. cerevisiae*, the rate-limiting enzyme for CoA synthesis) and the “acetyl-“part through PDH bypass introduction (*ALD6* from *S. cerevisiae* and Se*Acs* ^*L641P*^ from *Salmonella enteric*). A naringenin-producing reporter strain had been constructed to reflect cytosolic acetyl-CoA level as acetyl-CoA is the precursor of naringenin. It was found that PanK overexpression or PDH bypass introduction alone only led to a twofold or 6.74-fold increase in naringenin titer, but the combination of both (strain CENFPAA01) had resulted in 24.4-fold increase as compared to the control (strain CENF09) in the presence of 0.5 mM substrate *p*-coumaric acid. The supplement of PanK substrate pantothenate resulted in another 19% increase in naringenin production.

**Conclusions:**

To greatly enhance acetyl-CoA level in yeast cytosol, it is feasible to engineer both the “acetyl-” part and the “-CoA” part simultaneously. Insufficient CoA supply might aggravate acetyl-CoA shortage and cause low yield of target product.

**Electronic supplementary material:**

The online version of this article (doi:10.1186/s13068-017-0726-z) contains supplementary material, which is available to authorized users.

## Background

The continuous use of fossil fuels has led to environment change. In recent years, people are seeking alternative energy resource to replace traditional fossil fuels [1]. Microbial conversion of renewable feedstock into fuels and chemicals has been intensively investigated [2]. *Escherichia coli* and *Saccharomyces cerevisiae* (*S. cerevisiae*), the most popular microbial factories, have been engineered for the production of valuable products [3, 4]. Compared with *E. coli*, yeast has unique advantages, such as post-translational modifications, capacity of expressing complex enzymes like P450s, less possibility of potential phage contamination [5–7]. Thus, it has been engineered to utilize various feedstocks to produce natural products and biofuels [8, 9].

**Fig. 1:**
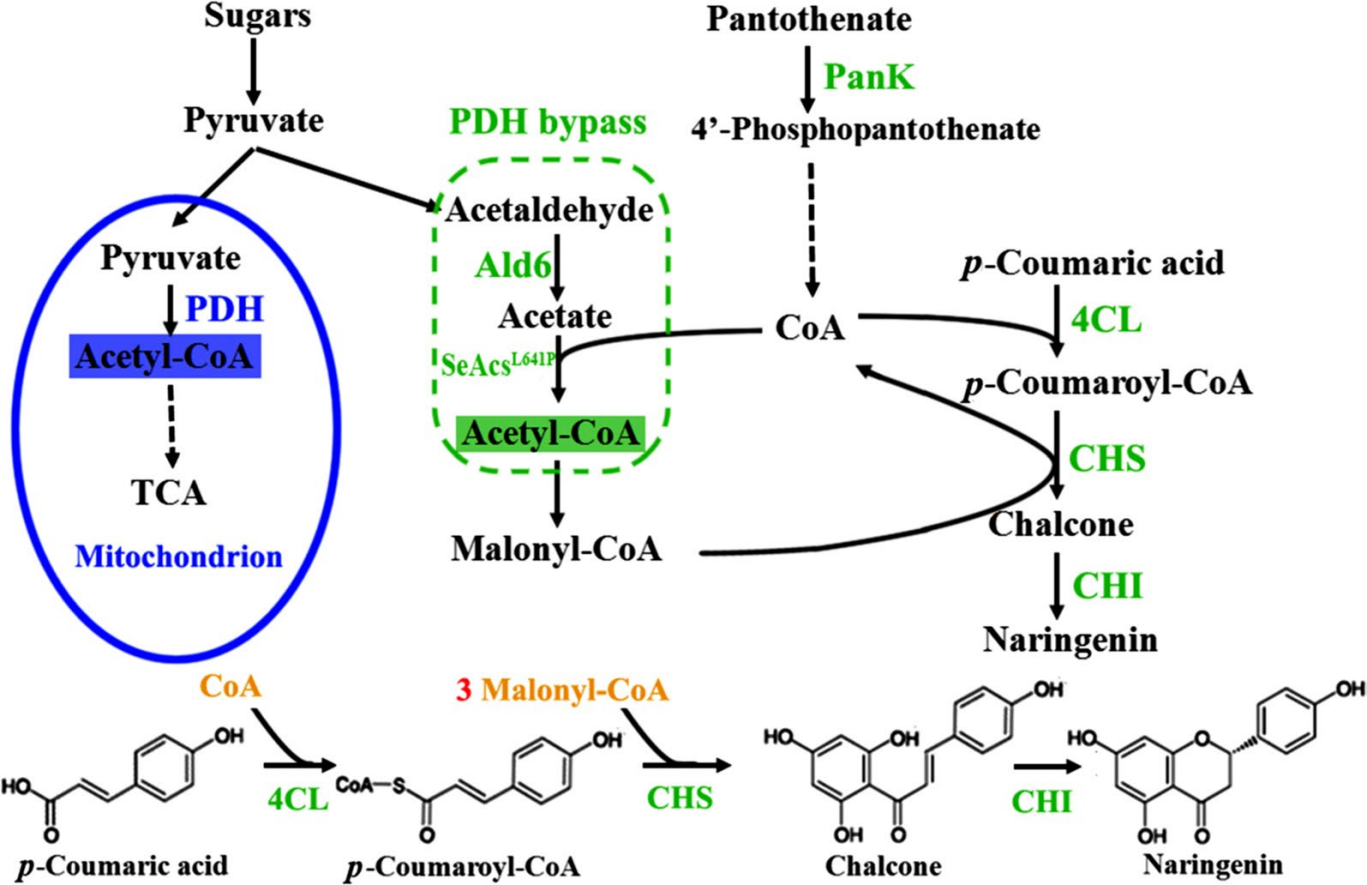
Simplified overview of acetyl-CoA metabolism and associated naringenin synthesis pathway in *S. cerevisiae*. Acetyl-CoA is generated in different compartments in yeast, including mitochondria (*blue*), cytosol [25], and peroxisome (not shown). Three genes (*4CL, CHS, and CHI*) for naringenin synthesis were introduced into yeast genome. In addition, the rate-limiting enzyme of CoA synthesis, pantothenate kinase (PanK), and PDH bypass (*ALD6* and Se*Acs* ^*L641P*^) were introduced into *S. cerevisiae* to improve its acetyl-CoA level in cytosol

Acetyl-CoA is the precursor of a wide range of bioproducts, including isoprenoids, polyketides, flavonoids, stilbenes, fatty acids and lipids, polyhydroxyalkanoates, and 1-butanol [1, 6, 10]. These products are mostly synthesized by consuming cytosolic acetyl-CoA. However, as acetyl-CoA in yeast is mainly generated in mitochondria from pyruvate through pyruvate dehydrogenase (PDH) complex, it needs carnitine/acetyl-carnitine shuttle to be transported out of mitochondria [6, 11]. In order to enhance acetyl-CoA level in cytosol, PDH bypass has been introduced into *S. cerevisiae* (Fig. 1). PDH bypass is composed of pyruvate decarboxylase (PDC), acetaldehyde dehydrogenase (ACDH, such as Ald6), and acetyl-CoA synthetase (ACS) [12]: the acetaldehyde formed from pyruvates by PDC can be converted into acetate by ACDH, or to ethanol by alcohol dehydrogenase (ADH), and acetyl-CoA can be generated from acetate by ACS. Specifically, endogenous *ALD6*, and endogenous *ACS1*/*ACS2* or Se*Acs* ^*L641P*^ from *Salmonella enteric* have been used in PDH bypass buildup [6, 13]. In addition, other strategies, such as overexpression of *ADH2* (ADH2 converts ethanol into acetaldehyde) [6, 13], knockout of *MLS1* and *CIT2* (encoding malate synthase and citrate synthase, respectively) [6], and knockout of *ADH1* to limit ethanol production from acetaldehyde [14], have been reported to enhance cytosolic acetyl-CoA supply in yeast. Combination of these approaches has been used to improve target product yield such as α-santalene [6]. But the combination may not always lead to significant increase in acetyl-CoA supply in yeast, for example, PDH bypass was introduced together with the overexpression of exogenous ATP-dependent citrate lyase (ACL) or PDH complex (cytosol), but little improvement was observed [7].

Previous studies on improving acetyl-CoA level in *S. cerevisiae* have been focused on engineering the “acetyl-” part of acetyl-CoA, but to the best of our knowledge, there have been no reports on engineering the “-CoA” part so far. Pantothenate kinase (PanK) is considered to be the rate-limiting enzyme for CoA synthesis, which catalyzes the phosphorylation of pantothenate [15] (Fig. 1). It was reported previously that the overexpression of *mPanK1β* (an isoform of PanK) in mammalian cells would trigger 13-fold increase in intracellular CoA content [16]. PanK overexpression in *E. coli* could also lead to tenfold increase in its intracellular CoA level and fivefold increase in its acetyl-CoA level [17]. Therefore, in this work, we tried to overexpress PanK encoding endogenous gene *CAB1* to increase acetyl-CoA level in *S. cerevisiae,* together with PDH bypass introduction (*ALD6* from *S. cerevisiae* and Se*Acs* ^*L641P*^ from *S. enteric*). In order to demonstrate cytosolic acetyl-CoA improvement in yeast, we had chosen naringenin as our model product, which takes acetyl-CoA as its precursor (Fig. 1). A naringenin pathway of three genes, i.e., 4-coumarate:CoA ligase (4CL), chalcone synthase (CHS), and flavanone isomerase (CHI), was integrated into yeast genome first. The introduction of PDH bypass alone led to 6.74-fold increase in naringenin titer in the presence of 0.5 mM substrate. PanK overexpression further enhanced naringenin production by 24.4-fold as compared to the control. The supplement of PanK substrate pantothenate resulted in another 19% increase in naringenin production. An independent acetyl-CoA assay also confirmed the enhancement in cytosolic acetyl-CoA level in the engineered yeast strains.

## Methods

### Plasmid and strain construction

PanK encoding gene *CAB1* was amplified from *S. cerevisiae* BY4742 genome using primer pair 1&2 (Table 1) with KAPA HIFI polymerase (KAPA Biosystems, Wilmington, MA, USA) and the PCR products were digested by *Spe*I and *Hind*III (all restriction enzymes in this study were from New England Biolab, Massachusetts, US) and inserted into the modified plasmid pRS426GAL1, which was reconstructed by removing its original *Xho*I and *Sal*I sites (a pair of isocaudamer). The truncated *HXT7* promoter amplified from yeast genome using primer pair 3 & 4 was digested by *Sac*I and *Spe*I and inserted between *Sac*I and *Spe*I sites to replace the original GAL1 promoter. The new plasmid was named p426PanK.

*TEF1p*-*ALD6*-*ADH1t* expression cassette (*TEF1p* promoter, *ALD6* and *ADH1t* terminator were all amplified from *S. cerevisiae* BY4742 genome) was constructed by overlap PCR using primers 5, 6, 7, 8, 9, and 10. Restriction endonuclease *Sac*I, *Bam*HI, *Xho*I, *Nhe*I, and *Not*I sites were introduced into the 5′-end of *TEF1p* using primer 5, while a *Sac*I site was introduced into the 3′-end of *ADH1t* using primer 10. The cassette was digested with *Sac*I and inserted into p426PanK to obtain plasmid p426PanK-Ald6. *TDH3p*-Se*Acs* ^*L641P*^-*PGIt* expression cassette, with *TDH3p* promoter and *PGIt* terminator from *S. cerevisiae* and Se*Acs* ^*L641P*^ from *Salmonella enteric*, was codon optimized and synthesized by GenScript (Piscataway, New Jersey, USA), which was digested by *Nhe*I and *Xho*I, and inserted into plasmid p426PanK-Ald6. The resulting plasmid was named p426PAA. The *TEF1p*-*ALD6*-*ADH1t* and *TDH3p*-Se*Acs* ^*L641P*^-*PGIt* expression cassettes were cut from plasmid p426PAA by *Sac*I and *Xho*I and inserted into plasmid pRS426Gal1 to generate plasmid p426AA. All constructed plasmids (Table 2) had been sent to DNA sequencing for confirmation.

**Table 1:** Primers in this study

Primer no.	Sequence (5′–3′)
1	GTCAACTAGTATGCCGCGAATTACTCAAGAG
2	GTACAAGCTTCTACGTACTTGTTTTCTTAGTAG
3	GTCAGAGCTCACTTCTCGTAGGAACAATTTC
4	GTCAACTAGTTTTTTGATTAAAATTAAAAAAAC
5	ATCTGAGCTCGGATCCACTCGAGAGCTAGCAGCGGCCGCCACACACCATAGCTTCAA
6	GTGTAGCTTAGTCATTTTGTAATTAAAACTTAG
7	AGTTTTAATTACAAAATGACTAAGCTACACTTTGAC
8	TCATAAGAAATTCGCTTACAACTTAATTCTGACAGC
9	AGAATTAAGTTGTAAGCGAATTTCTTATGATTTATG
10	CTATGAGCTCGATCCGTGTGGAAGAACG
11	TTGTAATCGTTCTTCCACACGGATCTGGGGCCGTATACTTACATAT
12	TGGAGCAACACAATCACCCATGTTTAGTTAATTATAGTTCGT
13	ACGAACTATAATTAACTAAACATGGGTGATTGTGTTGCTCCA
14	GTAAAGACATAAGAGATCCGCTTACTTTGGCAAATCACCAGA
15	TCTGGTGATTTGCCAAAGTAAGCGGATCTCTTATGTCTTTAC
16	AAACATTTTGAAGCTATGGTGTGTGGGCATGCGAAGGAAAATGAGA
17	GATGATAGTTGATTTCTATTCCAACAGTGAGTAAGGAAAGAGTGAGGAAC
18	AACAGAAACTGGTGGAGACATTGTTTTATATTTGTTGTAAAAAG
19	CTTTTTACAACAAATATAAAACAATGTCTCCACCAGTTTCTGTT
20	CATAAATCATAAGAAATTCGCTTAAACACCAATAACTGGAAT
21	ATTCCAGTTATTGGTGTTTAAGCGAATTTCTTATGATTTATG
22	TACTATATGTAAGTATACGGCCCCAGGATCCGTGTGGAAGAACGAT
23	TCTCATTTTCCTTCGCATGCCCACACACCATAGCTTCAAAATG
24	AAGGGTTGTCGACCTGCAGCGTAGCAAATTAAAGCCTTCGAGC
25	TGGGACGCTCGAAGGCTTTAATTTGCTACGCTGCAGGTCGACAAC
26	CAACAACACCTGCTTCATCAGCTGTTACGACTCACTATAGGGAGACCG
27	CTCGAGGGATATAGGAATCCTC
28	GTTCCTCACTCTTTCCTTACTCACTGTTGGAATAGAAATCAACTATCATC
29	CCGGTCTCCCTATAGTGAGTCGTAACAGCTGATGAAGCAGGTGT
30	GAGAACTTCTAGTATATTCTGTATACCTAATATT

**Table 2:** Plasmids in this study

Name	Description	Source
pRS426GAL1	(*2μ URA3*)	[13]
pUG6	Contains *loxP*-*KanMX*-*loxP* cassette for knockout in yeast	[37]
p426PanK	*P*_*HXT7*_-*CAB1* (*2μ URA3*)	This study
p426PanK-Ald6	*P*_*HXT7*_-*CAB1 P*_*TEF1*_-*ALD6* (*2μ URA3*)	This study
p426AA	*P*_*TEF1*_-*ALD6 P*_*TDH3*_-Se*Acs* ^*L641P*^ (*2μ URA3*)	This study
p426PAA	*P*_*HXT7*_-*CAB1 P*_*TEF1*_-*ALD6 P*_*TDH3*_-Se*Acs* ^*L641P*^ (*2μ URA3*)	This study

To construct a naringenin-producing reporter strain, three genes involved in naringenin synthesis pathway were introduced into *S. cerevisiae* genome, namely 4-coumarate:CoA ligase (*4CL*) cDNA (GenBank: X13324) from *Petroselinum crispum*, chalcone synthase (*CHS*) cDNA (GenBank: AF233638) from *Petunia hybrida*, and flavanone isomerase (*CHI*) cDNA (GenBank: Y00852) from *Petunia hybrida*. *4CL*, *CHI*, and *TEF1p*-*CHS*-*CYC1t* expression cassettes were codon optimized and synthesized by GenScript (an additional file shows this in more detail, see Additional file 1). *TEF2p*-*4CL*-*ADH2t* and *PGK1p*-*CHI*-*ADH1t* expression cassettes were constructed by overlap PCR using primers 11–16 and 17–22. *TEF1p*-*CHS*-*CYC1t* expression cassette was amplified by primer 23 & 24. To facilitate the integration, three additional DNA fragments were also obtained: *KanMX* (selective marker) was amplified from plasmid pUG6 using primer 25 & 26, δ1 (248 bp in the 5′-end of δ sequence) and δ2 fragments (239 bp in the 3′-end of δ sequence) were amplified from yeast genome using primer 27 & 28 and primer 29 & 30, respectively. These six DNA fragments were assembled into the δ sites of *S. cerevisiae* CEN.PK2-1C chrome (*MATa; ura3*-*52; trp1*-*289; leu2*-*3,112; his3 Δ1; MAL2*-*8C; SUC2*) by a DNA assembler method [18]. The successfully assembled strain (CENF09) was confirmed by PCR with aforementioned primers (Table 3). Plasmid p426PanK was transformed into strain CEN.PK2-1C and CENF09 to create CENP01 and CENFP01, respectively, using the LiAc/SS carrier DNA/PEG method [19]. The same approach was used to transform plasmid p426AA into strain CEN.PK2-1C and CENF09 to generate CENAA01 and CENFAA01, respectively. Plasmid p426PAA was also transformed into strain CEN.PK2-1C and CENF09 to obtain CENPAA01 and CENFPAA01, respectively. In order to identify positive clones, plasmids were extracted with Zymoprep Yeast Plasmid Miniprep II Kit (Zymo Research, Irvine, CA) and transformed into *E. coli* for verification by both restriction enzyme digestion and PCR.

**Table 3:** Strains in this study

Name	Description	Source
CEN.PK2-1C	*MATa; ura3*-*52; trp1*-*289; leu2*-*3,112; his3 Δ1; MAL2*-*8C; SUC2*	EUROSCARF
CENF09	CEN.PK2-1C with naringenin synthesis pathway (*P*_*TEF2*_-*4CL P*_*TEF1*_-*CHS P*_*PGK1*_-*CHI*) integrated into δ sites in chromosome, using *KanMX* for selection	This study
CENP01	CEN.PK2-1C + p426PanK	This study
CENFP01	CENF09 + p426PanK	This study
CENAA01	CEN.PK2-1C + p426AA	This study
CENFAA01	CENF09 + p426AA	This study
CENPAA01	CEN.PK2-1C + p426PAA	This study
CENFPAA01	CENF09 + p426PAA	This study

### Media and growth conditions

*Escherichia coli* DH5α was used for cloning and cultured in Luria–Bertani [20] broth with 100 μg/mL ampicillin at 37 °C. Yeast cells were cultured in YPD media (20 g/L peptone, 10 g/L yeast extract, and 20 g/L glucose) at 30 °C. Recombinant yeast strains were screened and grown in YPD containing 200 μg/mL G418, or auxotrophic Complete Minimal medium (CM, 6.7 g/L yeast nitrogen base without amino acids, 20 g/L glucose, 150 mg/L valine, 20 mg/L adenine hemisulfate, 20 mg/L arginine-HCl, 30 mg/L lycine-HCl, 20 mg/L methionine, 200 mg/L threonine, 30 mg/L tyrosine, 50 mg/L phenylalanine, optionally supplemented with 100 mg/L leucine, 20 mg/L histidine, 20 mg/L uracile, and 20 mg/L tryptophane) at 30 °C.

### Naringenin fermentation and HPLC analysis

Yeast colonies of CENF09, CENFP01, CENFAA01, and CENFPAA01 were pre-cultured in 5-mL CM medium in 50-mL tubes overnight at 30 °C, 225 rpm, respectively. The pre-culture was then diluted into fresh 20-mL CM medium in 250-mL flasks to a final OD_600_ of 0.05, respectively. Fermentation was carried out at 30 °C, 225 rpm for 96 h, with substrate *p*-coumaric acid (Sigma-Aldrich, St. Louis, MO, USA) concentration at 0.5 mM. In addition, the best acetyl-CoA-producing strain was also tested at a series of *p*-coumaric acid concentration: 0.05, 0.1, 0.2, 0.3, 0.4, and 0.5 mM.

The fermentation broth was centrifuged at 12,000 rpm for 10 min. Samples from each supernatant were taken for HPLC analysis on a XDB-C18 column (Agilent, Santa Clara, USA). Compounds were separated by elution with acetonitrile–water gradient at 1.0 ml/min as described previously [21]. Naringenin standard (ACROS organics, New Jersey, USA) and naringenin from the samples were detected by its UV absorbance at 290 nm.

### Acetyl-CoA measurement

Acetyl-CoA was analyzed according to a previously described method [7]. Yeast colonies of CENP01, CENAA01, and CENPAA01 and wild-type CEN.PK2-1C were pre-cultured in 5-mL CM medium in 50-mL tubes overnight at 30 °C, 225 rpm. The pre-cultures were diluted into fresh 50-mL CM medium to a final OD_600_ of 0.05. Cells were harvested during mid-log phase by centrifugation at 12,000 rpm for 5 min. 10-mL pre-chilled (−80 °C) methanol was added to quench cell metabolism and centrifuged at 12,000 rpm for 5 min to remove the supernatant. 2 mL boiling ethanol was added to cell pellets and the mixture was treated thoroughly by glass beads for 5 min (vortex) to release intracellular metabolites. The supernatant was vacuum dried after centrifugation and re-suspended in 200 μL ddH_2_O. The resulting solution containing acetyl-CoA was analyzed by an Acetyl-CoA Assay Kit (Sigma-Aldrich, St. Louis, MO, USA). Acetyl-CoA concentration obtained was an average of biological duplicates, normalized by dry cell weight.

### Pantothenate effect on naringenin production

CENFPAA01 was pre-cultured in 5 mL CM medium in 50-mL tubes overnight at 30 °C, 225 rpm. The pre-cultures were diluted into fresh 20 mL CM medium containing 0.5 mM *p*-coumaric acid substrate to a final OD_600_ of 0.05. 50 mM pantothenate (Sigma-Aldrich, St. Louis, MO, USA) stock solution was added to the liquid medium to achieve a final pantothenate concentration of 10, 20, and 50 μM, respectively. Fermentation was carried out at 30 °C, 225 rpm for 96 h. Samples were taken from each fermentation broth for naringenin measurement.

## Results

### Construction of naringenin-producing reporter strain

**Fig. 2:**
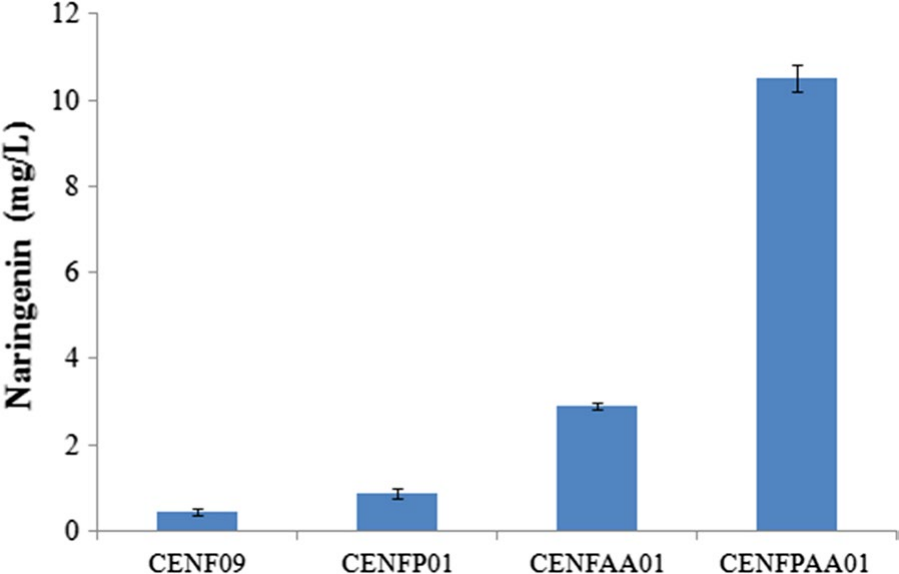
Naringenin titer in the engineered yeast strain CENF09 (the control), CENFP01, CENFAA01, and CENFPAA01 after 96 h fermentation in CM medium at 30 °C. *Error bars* represent the standard errors of at least three replicates

The amount of acetyl-CoA, an important precursor, is under dynamic changes due to continuous generation and consumption processes, and hence it is difficult to measure accurately [22, 23]. Since acetyl-CoA is an important precursor for naringenin, naringenin pathway was introduced into *S. cerevisiae* as a reporter of its cytosolic acetyl-CoA level. Naringenin can be synthesized from *p*-coumaric acid in plants, which is catalyzed by three enzymes, 4CL, CHS, and CHI (Fig. 1). 4CL catalyzes the ligation of CoA and *p*-coumaric acid to generate *p*-coumaroyl-CoA. CHS is responsible for the condensation of three molecules of malonyl-CoA, which are generated from 3 molecules acetyl-CoA, and *p*-coumaroyl-CoA to produce chalcone. Lastly, CHI catalyzes the isomerization of chalcone to generate naringenin. Three molecules of acetyl-CoA are consumed in total for one molecule of naringenin generated. In this work, in order to construct a stable reporter strain, *4CL* gene from *P. crispum*, *CHS* and *CHI* genes from *P. hybrida* were codon optimized and synthesized for DNA assembly. All three genes were integrated into yeast genome by a single transformation step to generate strain CENF09. Constitutive promoters *TEF1p*, *TEF2p*, and *PGK1p* were chosen for *CHS*, *4CL,* and *CHI* transcription, respectively. As a result, strain CENF09 could generate 0.43 mg/L naringenin with 0.5 mM *p*-coumaric acid present (Fig. 2).

### Enhancing CoA/acetyl-CoA supply with PanK overexpression

CoA was synthesized from pantothente, cysteine and ATP. For the first step, pantothenate is phosphorylated to 4′-phosphopantetheine by an ATP-dependent pantothenate kinase (Pank). 4′-phosphopantetheine reacts with cysteine to form 4′-phosphopantothenoylcysteine, which is subsequently decarboxylated to generate 4′-phosphopantetheine, which is changed into dephospho-CoA that is finally phosphorylated into CoA. The reaction catalyzed by pantothenate kinase is the key and rate-limiting step. PanK is encoded by CAB1 in *S. cerevisiae*, which is reported to be transcribed at low level [15]. To improve CoA synthesis, *CAB1* was overexpressed under a strong constitutive promoter (truncated *HXT7* promoter) in CENF09 to create strain CENFP01, and the naringenin titer was found to be 0.88 mg/L in the presence of 0.5 mM substrate, about two-fold that of the control CENF09 (Fig. 2).

### Introduction of PDH bypass to further improve acetyl-CoA level

A PDH bypass, which generates acetyl-CoA from acetaldehydes via Ald6 from *S. cerevisiae* and mutant ACS from *S. enteric* (SeAcs^L641P^), was reported previously to enhance acetyl-CoA supply for amorphadiene [13] and α-santalene [6]. Due to the difficulty to overexpress PDC complex, *ALD6* gene (*S. cerevisiae*) and Se*Acs* ^*L641P*^ (*S. enteric*) were overexpressed in this study for PDH bypass construction, under constitutive *TEF1* and *TDH3* promoter in plasmid p426AA, which was introduced into yeast to create strain CENFAA01. It had demonstrated better naringenin production—2.90 mg/L naringenin with 0.5 mM *p*-coumaric acid present, 6.74-fold increase as compared to that of the control CENF09 (Fig. 2).

In order to further improve intercellular acetyl-CoA level in yeast, PDH bypass and PanK were both introduced into CENF09 to generate strain CENFPAA01. A significant enhancement in naringenin titer was observed in CENFPAA01, 10.51 mg/L, which was 24.44-fold increase as compared to that of the control CENF09, 11.94-fold of CENFP01, and 3.63-fold of CENFAA01 (Fig. 2).

**Fig. 3:**
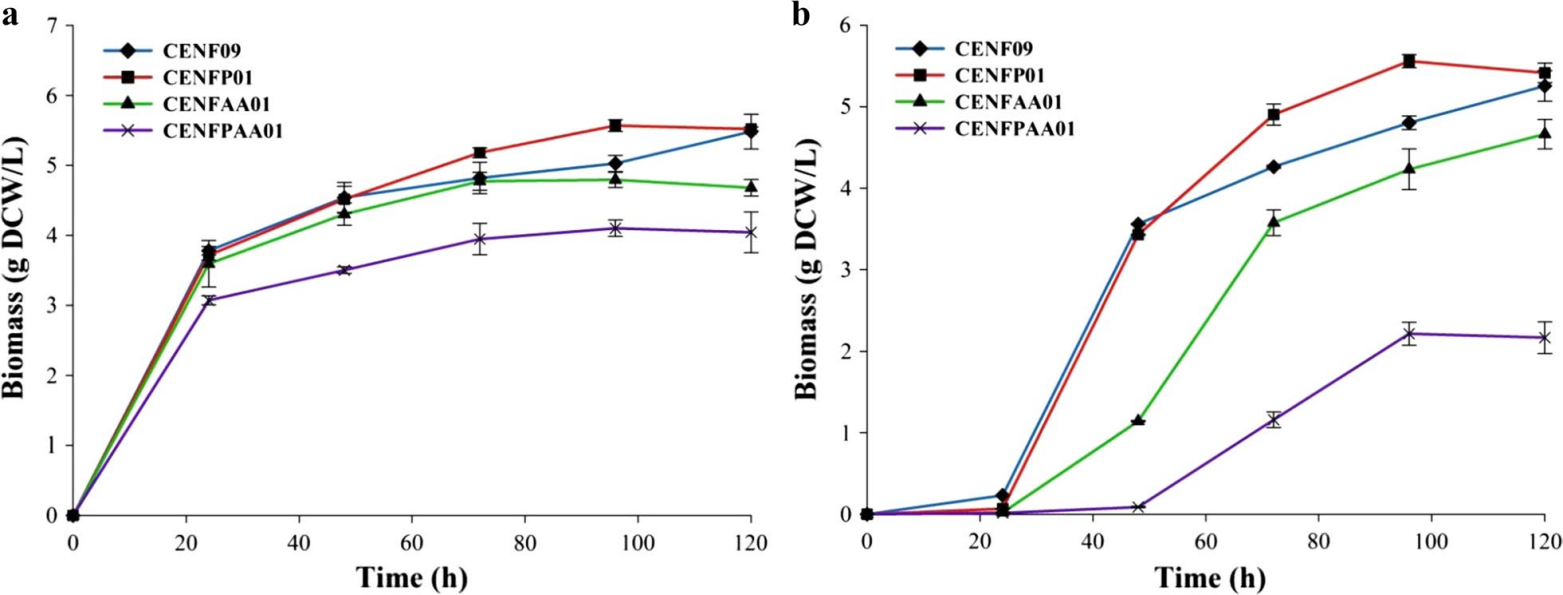
Growth profile of naringenin-producing strains in CM medium (**a**) without *p*-coumaric acid; **b** with 0.5 mM *p*-coumaric acid. *Error bars* represent standard errors of at least three replicates

We had also tracked cell growth of CENF09, CENFP01, CENFAA01, and CENFPAA0 during naringenin production. As shown in Fig. 3a, there is no significant differences on the growth after 120 h culture in CM medium among strain CENF09, CENFP01, and CENFAA01, whereas CENFPAA01 showed much less biomass accumulation, which means the introduction of PanK or PDH bypass alone did not affect cell growth very much, but the co-expression of PanK and PDH bypass had caused heavy metabolic burden to yeast cells. Substrate *p*-coumaric acid itself also demonstrated inhibitory effects on cell growth during the first 24 h as shown in Fig. 3b. *p*-coumaric acid almost did not change the final biomass of CENF09 and CENFP01 after 120 h, while CENFAA01 grew slower than without *p*-coumaric acid. Especially, CENFPAA01 in CM with 0.5 mM *p*-coumaric acid grew much slower than that of without *p*-coumaric acid. Moreover, if taking cell growth into account, naringenin titer in the best acetyl-CoA-producing strain CENFPAA01 was 54-fold that of CENF09, 30-fold of CENFP01, and sevenfold of CENFAA01, in the presence of 0.5 mM *p*-coumaric acid.

**Fig. 4:**
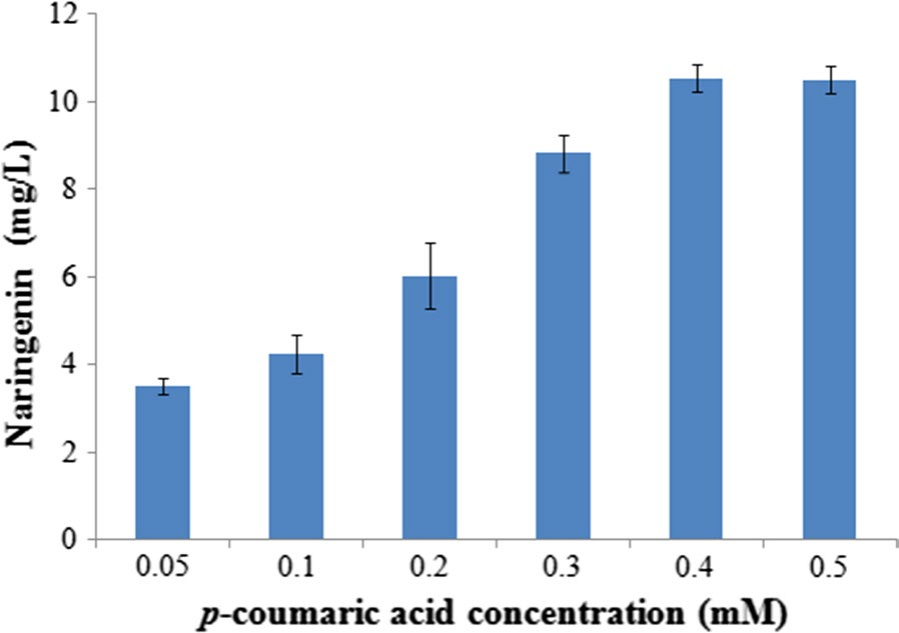
Naringenin production in CENFPAA01 with various substrate *p*-coumaric acid concentration, 96 h fermentation in CM medium at 30 °C. *Error bars* represent the standard errors of at least three replicates

Since CENFPAA01 was the best naringenin- and acetyl-CoA-producing strain obtained, it was utilized for substrate optimization. As shown in Fig. 4, naringenin production increased with *p*-coumaric acid concentration and reached its optimum (~10.50 mg/L) at 0.4–0.5 mM *p*-coumaric acid. As other research groups had also reported their naringenin production with 0.5 mM *p*-coumaric acid [21, 24, 25], we set substrate concentration at 0.5 mM in this work.

### Acetyl-CoA assay

**Fig. 5:**
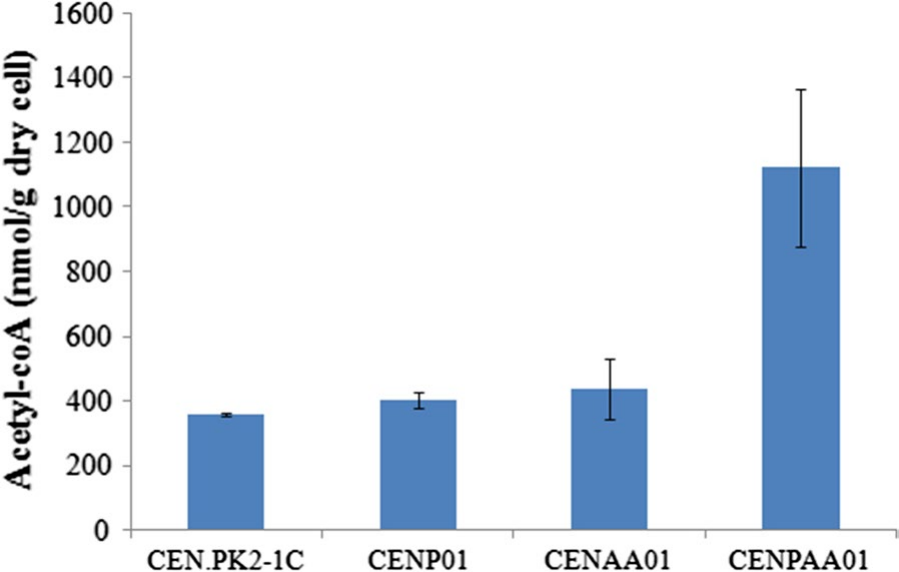
Acetyl-CoA level in the engineered yeast strain CENP01, CENAA01, and CENPAA01 after 96 h culture in CM medium at 30 °C, with native strain CEN.PK2-1C as control. Acetyl-CoA level was normalized by dry cell weight

To further confirm that the rise in naringenin production was due to acetyl-CoA increase in yeast, we had determined acetyl-CoA concentration in native strain CEN.PK2-1C and three non-naringenin-producing strains (CENP01, CENAA01, and CENPAA01) with Acetyl-CoA Assay Kit, of which a CoA quencher would help remove free CoA background. As displayed by Fig. 5, the overexpression of PanK (CENP01) or PDH bypass (CENAA01) alone did not show significant acetyl-CoA level improvement as compared to native strain CEN.PK2-1C. However, when PanK and PDH bypass were co-expressed in CENPAA01, acetyl-CoA level was greatly enhanced by ~threefold as compared to CEN.PK2-1C, which was in agreement with aforementioned findings on naringenin generation (Fig. 2).

### Pantothenate effect on intracellular acetyl-CoA level

**Fig. 6:**
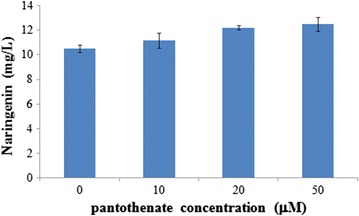
Pantothenate effect on naringenin production in CENFPAA01, 96 h fermentation in CM medium at 30 °C. *Error bars* represent the standard errors of at least three repeats. *PA* pantothenate

Pantothenate, the substrate for PanK and precursor for CoA, is directly related with acetyl-CoA biosynthesis in *S. cerevisiae*. Since PanK is the rate-limiting step for CoA synthesis and previous reports have shown that pantothenate supplement could help increase CoA/acetyl-CoA level in mammalian and *E. coli* cells [16, 17], pantothenate concentration in the fermentation broth was optimized in this work for naringenin production. As shown in Fig. 6, the titer rose with pantothenate addition when pantothenate concentration increased from 10 to 50 μM. Naringenin titer was at its maximum in the presence of 50 μM pantothenate (12.49 mg/L), just a bit higher than that of 20 μM (12.22 mg/L), ~19% increase as compared to the case without (10.51 mg/L), which also suggests that increasing CoA supply is crucial for improving cytosolic acetyl-CoA level in yeast.

## Discussion

In this work, PDH bypass and PanK, the rate-limiting enzyme for CoA synthesis, were both introduced into a naringenin-producing reporter strain to demonstrate its cytosolic acetyl-CoA level improvement. The best engineered strain CENFPAA01 showed naringenin titer at 10.51 mg/L in the presence of 0.5 mM *p*-coumaric acid, which was 24.4-fold increase of that of the control CENF09. PanK substrate, pantothenate supplement has led to another 19% increase in naringenin production, and the final titer was further increased to 12.49 mg/L, which suggests that enhancing CoA supply could help improve acetyl-CoA level in yeast.

We found that to greatly enhance acetyl-CoA level in yeast cytosol, both the “acetyl-” part and the “-CoA” part have to be engineered simultaneously. The introduction of PanK or PDH bypass alone only showed moderate enhancement in naringenin production, namely twofold and 6.74-fold, respectively. However, when both were introduced into strain CENFPAA01, naringenin titer was dramatically improved by 24.4-fold as compared to the control CENF09. These findings were double confirmed by an independent acetyl-CoA assay (Fig. 5). Previous studies on ACL and PDH complex overexpression have successfully improved target products with acetyl-CoA as precursors [26, 27]. However, ACL catalyzes the formation of acetyl-CoA and oxaloacetate from citrate and CoA in the presence of ATP in cytoplasm. PDH complex overexpression mainly focuses on increasing the acetyl- part. Thus, the supply of CoA might affect the final acetyl-CoA production. The idea is in agreement with previous findings that the introduction of PDH bypass alone could not improve cytosolic acetyl-CoA level greatly in yeast. Chen et al. showed that PDH bypass could only enhance α-santalene titer by 50% in *S. cerevisiae* [6, 11]. Compared with above methods, our method is to ensure the balance between CoA and acetyl- part and maximize the acetyl-CoA production. The “-CoA” engineering approach via PanK overexpression discussed here probably can also be combined with other “acetyl-” engineering methods to further help increase cytosolic acetyl-CoA supply in yeast.

The maximum naringenin titer reported here is 12.49 mg/L, better than the titer from a previous report of introducing phenylalanine ammonia lyase [28] for de novo synthesis of naringenin (5.8 mg/L) [29]. Our titer was still lower than the naringenin titer (28.3 mg/L) reported by Koffas group, which was achieved by adding substrate *p*-coumaric acid every 13 h to the culture in five equal doses [30]. To the best of our knowledge, all studies on flavonoid production in yeast used expression plasmids containing *GAL1* or *GAL10* promoter [29–31]. However, the repetitive homologous sequence of promoters may cause high possibility of gene deletion after rounds of subcultures [32, 33]. In this work, we had constructed a stable naringenin-producing strain for the evaluation of acetyl-CoA level in yeast—*4CL, CHS,* and *CHI* for naringenin synthesis were regulated by constitutive promoters and integrated into yeast genome.

Yan et al. [34] added substrate *p*-coumaric acid every 13 h to avoid its toxicity to yeast cells. Interestingly, we had also found that the control CENF09 had higher naringenin production at low *p*-coumaric acid concentration, namely 3.55 mg/L with 0.1 mM substrate and 0.43 mg/L with 0.5 mM. One possible explanation for this phenomenon could be the insufficient acetyl-CoA supply when *p*-coumaric acid concentration increased from 0.1 to 0.5 mM. CHS was reported to be the rate-limiting enzyme for flavonoid synthesis in oat primary leaves [34], and the flavonoid production could be regulated by CHS expression in *Juglans nigra* and cucumber plants [35, 36]. As such, if acetyl-CoA supply is insufficient, CHS reaction would slow down and less naringenin would be generated. At the same time, larger amount of CoA is consumed to produce *p*-coumaroyl-CoA with *p*-coumaric acid concentration increases from 0.1 to 0.5 mM. Hence, insufficient CoA supply might aggravate acetyl-CoA shortage for the CHS step and led to lower naringenin titer.

## Conclusions

In this study, we have demonstrated that the combination of PDH bypass and PanK overexpression would greatly enhance acetyl-CoA level in *S. cerevisiae* cytosol. It is the first report to engineer both the “acetyl-” part and the “-CoA” part simultaneously in yeast to improve acetyl-CoA production. Taking naringenin as sample product, the acetyl-CoA increase has led to 24.4-fold increase in its titer. We hope this approach could also help improve other chemical production in yeast, which takes acetyl-CoA as its precursor.

## Additional file

**Additional file 1.** Additional materials.

## Abbreviations

PanK: pantothenate kinase
PDH: pyruvate dehydrogenase
PDC: pyruvate decarboxylase
ALD6: acetaldehyde dehydrogenase
ACS: acetyl-CoA synthetase
ADH: alcohol dehydrogenase
4CL: 4-coumarate:CoA ligase
CHS: chalcone synthase
CHI: flavanone isomerase
