# Supplementary material for: Improving acetyl-CoA biosynthesis in Saccharomyces cerevisiae via the overexpression of pantothenate kinase and PDH bypass
Source: Biotechnol Biofuels. 2017 Feb 17;10:41. doi: 10.1186/s13068-017-0726-z (PMC5316175; doi:10.1186/s13068-017-0726-z)
Supplement: Supplementary file 1 — Additional file 1. Additional materials. [file 13068_2017_726_MOESM1_ESM.docx]

**Improving acetyl-CoA biosynthesis in *Saccharomyces cerevisiae* *via* the overexpression of pantothenate kinase and PDH bypass**

**Supplemental Materials**

**CHI sequences after optimization:**

atgtctccaccagtttctgttaccaagatgcaagttgaaaattacgctttcgctccaactgttaatccagctggttctactaatactttgtttttggctggtgctggtcatagaggtttggaaattgaaggtaaattcgttaagttcaccgccatcggtgtttacttggaagaatctgctattccattcttggctgaaaaatggaaaggtaagactccacaagaattgaccgattccgttgaattcttcagagatgttgttactggtccattcgaaaagttcactagagttaccatgattttgccattgaccggtaagcaatactctgaaaaggttgctgaaaactgtgttgctcattggaagggtattggtacttacactgatgatgaaggtagagctattgaaaagttcttggacgttttcagatccgaaacttttccaccaggtgcttctattatgttcactcaatctccattgggtttgttgaccatttctttcgctaaggatgattccgttactggtactgctaatgccgttattgaaaacaagcaattgtccgaagctgtcttggaatccattattggtaaacatggtgtttctccagctgctaaatgttctgttgcagaaagagttgccgaattattgaagaagtcttacgctgaagaagcctctgtttttggtaaaccagaaaccgaaaagtccaccattccagttattggtgtttaa

**4CL sequences after optimization:**

atgggtgattgtgttgctccaaaagaagatttgattttcagatccaagttgccagacatctacattccaaaacatttgccattgcatacctactgcttcgaaaacatttctaaggttggtgacaagtcctgcttgattaacggtgctactggtgaaactttcacttactcccaagtcgaattattgtccagaaaagttgcttccggtttgaacaagttgggtattcaacaaggtgataccatcatgttgttgttgccaaattctccagaatacttcttcgcttttttgggtgcttcttacagaggtgctatttctactatggctaatccatttttcacctccgccgaagttattaagcaattgaaagcttctttggccaagttgattattacccaagcttgctatgttgacaaggttaaggattatgctgccgaaaagaacatccaaatcatctgcattgatgatgctccacaagattgcttgcatttctctaaattgatggaagccgacgaatctgaaatgccagaagttgttattgattccgatgatgttgttgctttgccatactcttcaggtactactggtttgccaaaaggtgttatgttgactcataagggtttggttacatccgttgctcaacaagttgatggtgataatccaaacttgtacatgcactccgaagatgttatgatctgcattttgcctttgttccacatctactcattgaacgctgttttgtgttgtggtttgagagctggtgttaccattttgatcatgcaaaagttcgatatcgtcccattcttggaattgatccaaaagtacaaggttaccatcggtccatttgttcctccaatagttttggctattgctaagtctccagttgtcgataagtacgatttgtcctctgttagaactgttatgtctggtgctgctccattgggtaaagaattggaagatgctgttagagctaagttcccaaatgctaaattgggtcaaggttacggtatgactgaagctggtccagttttagctatgtgtttggcatttgctaaagaaccatacgaaatcaagtcaggtgcttgtggtactgttgttagaaatgctgaaatgaagatcgtcgacccagaaactaatgcttcattgccaagaaatcaaagaggtgaaatctgcatcagaggtgaccaaattatgaagggttacttgaacgatccagaatctactagaaccaccattgatgaagaaggttggttgcatactggtgatattggtttcattgatgacgacgacgaattattcatcgttgatagattgaaagaaatcatcaagtacaaaggtttccaagtcgctccagctgaattagaagctttgttgttaactcatccaaccatttctgatgctgctgttgttccaatgattgacgaaaaagctggtgaagttccagttgcttttgttgtcagaactaacggtttcactactaccgaagaagaaatcaaacaattcgtttccaagcaagtcgttttctacaagagaatcttcagagttttcttcgttgatgccattccaaagtctccatctggtaagattttgagaaaagacttgagagccaaaatcgcctctggtgatttgccaaagtaa

**CHS sequences after optimization:**

atggtcaccgtcgaagaatacagaaaagctcaaagagctgaaggtccagctactgttatggctattggtactgctactccaactaactgtgttgatcaatctacttacccagactactacttcagaatcactaactctgaacacaagaccgacttgaaagaaaagttcaagagaatgtgcgaaaagtccatgatcaagaaaagatatatgcacttgaccgaagaaatcttaaaagaaaacccatccatgtgcgaatatatggctccatctttggatgctagacaagatatcgttgttgttgaagttccaaagttgggtaaagaagctgctcaaaaggctatcaaagaatggggtcaacctaagtctaagatcacccatttggttttctgtactacctctggtgttgatatgccaggttgtgattatcaattgaccaagttgttgggtttaagaccatccgttaagagattgatgatgtaccaacaaggttgttttgctggtggtactgttttgagattggctaaagatttggccgaaaacaacaaaggtgctagagttttggttgtctgctctgaaattactgctgttacttttagaggtccaaacgatactcacttggattctttggttggtcaagctttgtttggtgatggtgctggtgctattatcattggttctgatccaattccaggtgtcgaaagaccattattcgaattggtttctgctgcccaaactttgttgccagattctcatggtgcaattgatggtcatttgagagaagttggtttgaccttccatttgttgaaagatgtcccaggtttgatttccaagaacatcgaaaagtctttggaagaagctttcagaccattgtccatctctgattggaattccttgttttggattgctcatccaggtggtccagcaattttggatcaagttgaaatcaagttgggtttgaagccagaaaagttgaaggctactagaaacgtcttgtctaactacggtaatatgtcctctgcttgcgttttgttcattttggacgaaatgagaaaggcctctgctaaagaaggtttgggtactactggtgaaggtttagaatggggtgttttatttggttttggtccaggtttgactgttgaaactgttgtcttgcattctgttgctacctga
